# Microbial community assembly, theory and rare functions

**DOI:** 10.3389/fmicb.2013.00068

**Published:** 2013-05-01

**Authors:** Mujalin K. Pholchan, Joana de C. Baptista, Russell J. Davenport, William T. Sloan, Thomas P. Curtis

**Affiliations:** ^1^Division of Biotechnology, Faculty of Science, Maejo UniversityChiangMai, Thailand; ^2^School of Civil Engineering and Geosciences, Newcastle UniversityNewcastle upon Tyne, UK; ^3^Department of Civil Engineering, Glasgow UniversityGlasgow, UK

**Keywords:** endocrine-disrupting chemicals, steroidal estrogens, theories of microbial community assembly, wastewater treatment

## Abstract

Views of community assembly have traditionally been based on the contrasting perspectives of the deterministic niche paradigm and stochastic neutral models. This study sought to determine if we could use empirical interventions conceived from a niche and neutral perspective to change the diversity and evenness of the microbial community within a reactor treating wastewater and to see if there was any associated change in the removal of endocrine disrupting compounds (EDCs). The systematic removal of EDCs and micropollutants from biological treatment systems is a major challenge for environmental engineers. We manipulated pairs of bioreactors in an experiment in which “niche” (temporal variation in resource concentration and resource complexity) and “neutral” (community size and immigration) attributes were changed and the effect on the detectable diversity and the removal of steroidal estrogens was evaluated. The effects of manipulations on diversity suggested that both niche and neutral processes are important in community assembly. We found that temporal variation in environmental conditions increased diversity but resource complexity did not. Larger communities had greater diversity but attempting to increase immigration by adding soil had the opposite effect. The effects of the manipulations on EDC removal efficiency were complex. Decreases in diversity, which were associated with a decrease in evenness, were associated with an increase in EDC removal. A simple generalized neutral model (calibrated with parameters typical of wastewater treatment plants) showed that decreases in diversity should lead to the increase in abundance of some ostensibly taxa rare. We conclude that neither niche and neutral perspectives nor the effect of diversity on putative rare functions can be properly understood by naïve qualitative observations. Instead, the relative importance of the key microbial mechanisms must be determined and, ideally, expressed mathematically.

## INTRODUCTION

Engineers create naturally assembled microbial communities in engineered systems that render ecosystem services for the use and benefit of mankind. The development of working engineered systems has typically run far ahead of microbial ecology with engineers using a mixture of trial and error and Monod kinetics to develop and design processes (Curtis et al., 2003). However, this approach has limitations and is subject to diminishing returns that can make it difficult for engineers to transcend current practices. Microbial ecology has, on occasion, led to the development of novel processes (van der Star et al., 2011). However, to break new ground and reliably engineer new functions, we believe it is necessary to understand what controls microbial diversity and the relationships between diversity and function. The rules that govern engineered systems should be the same as those that govern natural ones, for the microbe is unaware of the distinction. Consequently, ample inspiration for those seeking those rules in natural or engineered microbial systems can be found in the classical ecological literature, which is rich in theory.

With respect to community assembly, microbial ecologists can borrow from two opposing, but not mutually exclusive, perspectives. Classically, microbial assembly processes have been considered to be deterministic in which the presence of microbes reflects the presence or absence of that organism’s niche (Chase and Leibold, 2003). Microbial ecologists routinely and un-controversially interpret their work in this context. Alternatively, one can view the formation of a community as a stochastic process in which microbes colonize an environment and change within it at random (Bell, 2001; Hubbell, 2001). While the more recent introduction of the stochastic or neutral community models (NCM) in general ecology have been controversial, in essence, they are reworkings of the widely accepted theory of island biogeography (MacArthur and Wilson, 1967). Neutral theory has been successfully applied to microbial communities (Sloan et al., 2006, 2007), including the successful prediction of a taxa volume curve (Woodcock et al., 2007), a central feature of the theory of island biogeography. In reality, the two perspectives are not mutually exclusive because birth, death, and immigration, which are central to NCM, are ineluctable features of microbial life whose rates are affected by differences between species. Indeed, niche and neutral processes have been combined in a dynamic microbial NCM describing a Californian wastewater treatment system (Ofiteru et al., 2010).

The relationship between diversity and function is also important. Many classical ecologists assert that higher levels of diversity promote improved ecosystem functioning (Tilman et al., 1997; Hooper et al., 2005). In naturally occurring microbial communities, evenness and the number of taxa are confounded (a more even distribution means more taxa are observed; Curtis et al., 2006). Two important studies have used pure cultures so that the findings with respect to richness (the number of taxa) and evenness, and the co-occurrence of specific taxa are not confounded. Bell et al. (2005) used artificial communities to demonstrate that greater taxonomic richness led to greater productivity in microcosms degrading leaf litter. (Curtis et al., 2006). Wittebolle et al. (2009) were able to hold diversity constant whilst manipulating evenness to establish that uneven communities exhibited reduced functionality and less resistance to stress. However, the range of taxa used in these highly controlled studies is far below those found in real microbial communities. Less controlled studies have suggested that the benefits of increasing diversity plateau relatively quickly and that most of the extraordinary diversity in microbial communities is redundant (Franklin and Mills, 2006).

Nevertheless, it is also possible that the rare taxa may indeed have important functions. In particular, they may be responsible for the degradation of low concentrations of environmentally or economically important chemicals. For example, rare microbes may be involved in the metabolism of estrogen, compounds that disrupt the endocrine systems of animals and aquatic biota, leading to abnormalities in reproductive structure and function. There are two major types of estrogens; (i) natural estrogens, i.e., 17-estradiol (E2), estrone (E1), and estriol (E3), which are mainly excreted from humans and mammals; and (ii) synthetic estrogens, such as 17 α-ethinyloestradiol (EE2), which is the main ingredient in oral contraceptive pills. Both synthetic and natural forms occur in wastewater and their removal in biological treatment systems is poorly understood and highly unpredictable (Sumpter, 2009). The relative abundance of taxa responsible for the degradation of even the best removed estrogens, e.g., E1, are indeed rare, representing less than 2% of the microbial community (Zang et al., 2008; Thayanukul et al., 2010).

There have been various attempts to explain differences in estrogen removal with respect to differences in the design and operational parameters of biological treatment systems (Vader et al., 2000; Joss et al., 2004; Clara et al., 2005; Koh et al., 2008; McAdam et al., 2010). These studies infer, but do not demonstrate, that competitive niche mechanisms govern this function and stochastic mechanisms have not been considered.

A deeper understanding of the relationship between community assembly and rare functions would permit us to control endocrine disrupting compound (EDC) removal in engineered biological systems and predict the fate of micropollutants in natural microbial systems. Meeting this challenge is both intrinsically interesting and economically important. We have sought to address this by using both niche and neutral concepts to manipulate the microbial diversity of a reactor and evaluating the associated changes in EDC removal in a partial factorial designed experiment. The putatively neutral interventions were the organic loading rate and the addition of soil. The loading rate controls the number of individuals, more individuals should mean more diversity. Adding soil should increase the immigration rate, and thus the diversity. The putative niche interventions were the range of sugars in the feed and temporal variation in feed concentration associated with batch feeding. We reasoned that feed with more sugars would have more niches as would the change in feed concentration in time. We could not unequivocally support simple hypotheses about the relationship between niche or neutral effects and diversity and function. Both approaches could increase or decrease diversity and increases in diversity did not always lead to increases in EDC removal. We found that the counter intuitive results can be interpreted in the context of neutral theory.

## MATERIALS AND METHODS

The experimental design employed has been described in detail by Pholchan et al. (2010), where the effect of microbial community diversity on the engineering functions of carbon and nitrogen removal was addressed. Briefly, a two level fractional factorial design was undertaken, which required the performance of eight experimental runs. We sought to change the diversity of the reactors by manipulating the following variables: types of feed (simple, a single sugar, and complex, multiple sugars), types of feeding regime (sequencing batch reactor, or SBR, and completely stirred tank reactor, or CSTR), organic loading rate (low and high) and the addition of exogenous bacteria to promote immigration (by addition of soil and non-addition of soil). The factorial analysis permitted the determination of the joint effects of these four independent parameters as: the strength of the effects, coefficients, standard deviation of coefficients and associated probabilities. The responses in this study were expressed as percentages of EDC removal, whilst the microbial diversity was tentatively inferred from the number of bands on denaturing gradient gel electrophoresis (DGGE) gels. The data obtained were analyzed using Minitab (version 14, Minitab Inc., USA), based on the analysis of variance (ANOVA).

Two laboratory-scale SBR and two CSTR reactors were constructed each with a working volume of 17 l and fed with synthetic sewage with two types of carbon source (single carbon source/complex carbon source) at two different strengths (600/1,200 mg chemical oxygen demand (COD/l). The carbon sources (mg/l) were, for the simple waste, glucose (600) and for the complex waste: glucose (78.1), sucrose (148), lactose (148), fructose (78.1), and starch (12.7). The concentration of each sugar used in the simple and complex carbon source was calculated to render a total COD of 600 mg/l. The remaining constituents, in mg/l, were peptone (10.0), KH_2_PO_4_ (40.0), NH_4_Cl (153), CaCl_2_ (13.9), MnSO_4_ (0.28), ZnCl_2_ (0.21), CuSO_4_ (0.25), and MgCl_2_ (36.2). The energy calculations were based on the bomb-calorimeter data of Southgate (1981). Soils used in this study were collected from the Rivington series at the University of Newcastle’s Cockle Park Farm (Northumberland, UK). To separate indigenous bacteria from soil, plant roots were removed and soils (100 g per reactor) were suspended in 250 ml of sterile distilled water with sodium cholate (0.1% (w/v)). The soil suspension was shaken for 15 min with glass balls (35 × 45 mm, BHD Chemical Ltd., England) before being placed into an ultrasonicator for 1 min. The solution was sieved and the dislodged cells were separated from soil particles by centrifuging at 500 × *g* for 1 min. Bacterial suspensions (150 ml) were added to each reactor daily. This is equivalent to adding ~6 × 10^6^ bacterial cells per ml or about 1–5% of the bacterial total count. The seeded soil organisms were calculated to be below the detection limit for denaturing gel electrophoresis (Muyzer et al., 1993).

The influent and effluent were collected every three days for physical and chemical analysis. Physical and chemical tests (i.e., pH, temperature, Alkalinity, COD, total organic carbon (TOC), total kjeldahl nitrogen (TKN), ${\mathrm{NO}}_{2}^{-}$ , ${\mathrm{NO}}_{3}^{-}$ , ${\mathrm{PO}}_{4}^{3-}$ , mixed liquor suspended solids (MLSS), mixed liquor volatile suspended solids (MLVSS), SV_30_, sludge volume index (SVI), and capillary suction time (CST)) were conducted according to the Standard Methods (Clesceri et al., 1998). A biomass sample was taken from the aeration tank of each reactor every week, preserved immediately in 50% ethanol and stored at -20°C prior to the molecular analysis. Changes in the microbial community were monitored by performing DGGE of polymerase chain reaction (PCR)-amplified 16S rRNA gene segments from the bacterial community. The method is described in detail elsewhere (Pholchan et al., 2010). DNA was extracted using the Fast-DNA SPIN for soil kit (Q-Bio gene, Cambridge, UK). The total bacterial community was analyzed using PCR amplification with the classical primers 2 and 3, which contained a GC clamp (Muyzer et al., 1993). DGGE bands were visualized under an UV transilluminator with the program Quantity One (Bio-Rad) and DGGE banding patterns were analyzed using the Bionumerics software (Applied Mathematics) and PAST statistical software (Hammer and Harper, 2006).

The initial concentration of each estrogen studied (i.e., E1, E2, E3, and EE2) in the feed was set to an equivalent of 1 μg/l in all experiments. This is a typical concentration found in the wastewaters in processes that combine returned water from the sludge dewatering process with the raw wastewater (Matsui et al., 2000) and has been used in previous laboratory-scale studies (Li et al., 2005). Estrogens E1, E2, and EE2 (Sigma-Aldrich, UK), were separately dissolved in acetone to the concentration of 1.0 mg/ml and stored at -20°C. Prior to dosing the feed, a mixed solution of all estrogens was prepared by diluting the stock solutions in distilled water to the desired concentration. The EDCs concentrations were measured by gas chromatography mass spectrometry (GC/MS).

The method used to determine concentrations of estrogens was modified by Pholchan et al. (2008) from the method originally described by Nakamura et al. (2001). Mirex (Sigma-Aldrich, UK) was used as an internal standard along with 17α estradiol (Sigma-Aldrich, UK), which was used as a surrogate standard. The method comprised solid phase extraction and derivatization before analysis using a Hewlett–Packard 6890 GC split/splitless injector (260°C) linked to a Hewlett–Packard 5973 Mass Spectrometer with negative-ion chemical ionization mode. All reagents (GC-MS grade) and chemical standards were supplied by Sigma-Aldrich, UK.

Before the estrogens were added, the reactors were all inoculated with 4 l of sludge from the aeration tank of a municipal wastewater treatment plant (Tudhoe Mill Wastewater Treatment Plant, Spennymoor, Durham). At the start of each experimental run, the reactors were re-inoculated in a similar fashion. The Tudhoe Mill wastewater treatment plant was designed to achieve both organic carbon and nitrogen removal. Immediately after delivery to the laboratory, the sludge was kept aerated before being seeded into the reactor within 1–2 h. The MLSS and MLVSS of the seed were approximately 3050 and 2130 mg/l, respectively. Each experimental run lasted for approximately 60 days from the start of the addition of 1μg/l estrogens to the feed. All reactors were operated at room temperature (20 ± 2°C). The pH of the mixed liquor was maintained between seven and eight. The reactors were aerated, to ensure that the dissolved oxygen concentration was ≥2 mg/l. A sludge age of 10 days was maintained for all reactors by wasting excess sludge four times per day.

Community assembly was modeled as described previously (Sloan et al., 2007) using a simple neutral model executed in Matlab, where the source term, Θ, was set to 176 to illustrate lower diversity and 1760 to illustrate higher diversity. The immigration parameter (*m*) was set to 3.2 × 10^-9^ and the total number of individuals in the reactors (*N*_*T*_) was set to 1.7 × 10^13^ (equivalent to 17 l with 10^9^ individuals/ml). The model is illustrative as we could not parameterise the model using the data available. The parameters for the higher diversity and immigration were chosen on the basis of earlier simulations of wastewater treatment plants (Curtis et al., 2006). The lower source diversity was chosen arbitrarily to illustrate the effect of low diversity clearly. In reality the putative difference in diversity would have been less pronounced.

## RESULTS

**FIGURE 1:**
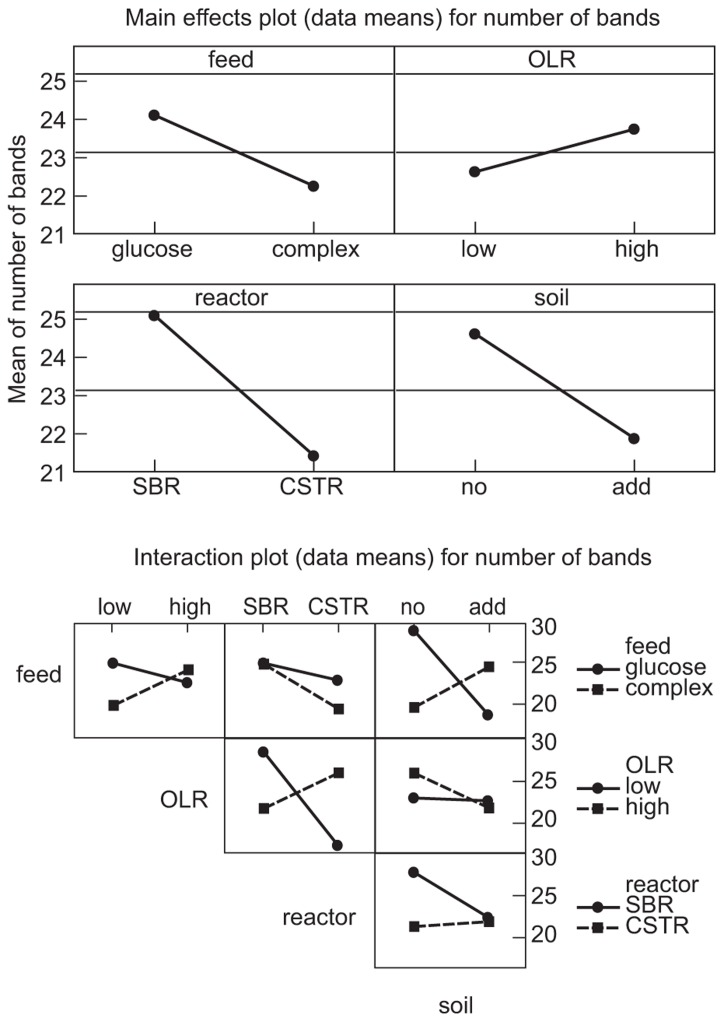
**Main effects plots (data means) on the number of bands: OLR, organic loading rate; SBR, sequencing batch reactor; CSTR, completely stirred tank reactor; no, no added soil; add, soil added to reactor**.

The manipulations had explicit effects on bacterial diversity: every intervention had a statistically significant effect on the number of bands detected (**Table 1; Figure 1**). However, the effects themselves were often counterintuitive. As expected, an increased organic load (more biomass) was associated with an increase in the diversity. Similarly a SBR (with temporal gradients as organic carbon and ammonia are utilized during the batch process) had more diversity than a homogenous stirred tank reactor. However, the use of more sugars in the feed (more nutritional complexity, more niches) was associated with a decrease in the observed richness. Similarly, the addition of soil (putatively increasing immigration) was associated with a decrease in the number of bands detected. The rather complex picture of the main effects is further complicated by the significant interactions between the experimental variables (**Table 1; Figure 1**). For example, the effect of the reactor format seemed to be partially dependant on the organic loading rate (**Table 1; Figure 1**).

**Table 1:** Calculated direction and significance of the main effects and their interactions on number of bands.

Term	Effect	Coefficient	SE of coefficient	*P* value
Constant		23.188	0.2421	0.000
Main factors				0.000
Type of carbon source	-1.875	-0.937	0.2421	0.005
Organic loading rate	1.125	0.563	*0.2421*	*0.049*
Type of feeding regime	-3.625	-1.813	0.2421	0.000
Adding soil	-2.625	-1.313	0.2421	0.001
Interaction of two factors				0.000
Type of carbon source × organic loading rate	3.375	1.688	0.2421	0.000
Type of carbon source × type of feeding regime	-1.875	-0.937	0.2421	0.005
Type of carbon source × adding soil	8.125	4.062	0.2421	0.000
*S* = 0.9682	*R*^2^ = 0.9826		*R*^2^_(adj)_ = 0.9673	

**FIGURE 2:**
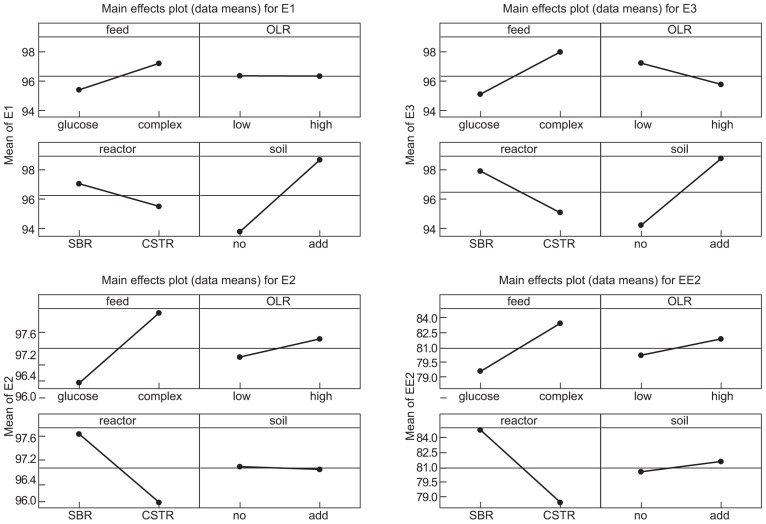
**Main effects plots (data means) on the removal of endocrine disrupting compounds**.

The interventions did have statistically significant effects on the removal of the EDCs (**Table 2; Figure 2**). However the effects were inconsistent between compounds and incongruent with our expectations about diversity and function. Thus adding soil, which apparently decreased putative richness, appeared to increase the removal of E1 and E3 and had no effect on E2 and EE2. Likewise the more complex feeds, which also ostensibly decreased the number of detectable bands, apparently increased the removal of E1, E2, and E3, but not EE2. By contrast the increase in diversity associated with an increased organic loading rate coincided with a decrease in E3 removal and had no change in the removal of E1, E2, and EE2. Only in the case of the SBR was the increased diversity associated with an increase in the removal of all the steroidal estrogens tested.

**Table 2:** Calculated direction and significance of the main effects and their interactions on the % removal of EDC in the reactors.

Term	Effect	Coefficient	SE of coefficient	*P* value
**A: estrone (E1)**
Constant		96.2750	0.3311	0.000
Main factors				0.001
Type of carbon source	1.7250	0.8625	0.3311	0.031
Organic loading rate	-0.050	-0.0250	0.3311	0.942
Type of feeding regime	-1.500	-0.7500	0.3311	0.053
Adding soil	*4.9750*	*2.4875*	0.3311**	*0.000*
Interaction of two factors				0.095
Type of carbon source × organic loading rate	0.9250	0.4625	0.3311	0.200
Type of carbon source × type of feeding regime	-0.7250	-0.3625	0.3311	0.305
Type of carbon source × adding soil	-1.6000	-0.8000	0.3311	0.042
*S* = 1.3243	*R*^2^^=^ 0.9063		*R*^2^_(adj)_^=^ 0.8243	
**B: 17-beta-estradiol (E2)**
Constant		96.8188	0.1851	0.000
Main factors				0.002
Type of carbon source	1.7625	0.8821	0.1851	0.001
Organic loading rate	0.4625	0.2313	0.1851	0.247
Type of feeding regime	-1.7125	-0.8563	0.1851	0.002
Adding soil	-0.0625	-0.0313	0.1851	0.870
Interaction of two factors				0.043
Type of carbon source × organic loading rate	-1.2625	-0.6313	0.1851	0.009
Type of carbon source × type of feeding regime	-0.4375	-0.2188	0.1851	0.271
Type of carbon source × adding soil	-0.0875	-0.0437	0.1851	0.819
*S* = 0.7404	*R*^2^^=^ 0.8801		*R*^2^_(adj)_^=^ 0.7753	
**C: estriol (E3)**
Constant		96.494	0.2292	0.000
Main factors				0.000
Type of carbon source	2.863	1.431	0.2292	0.000
Organic loading rate	-1.463	-0.731	0.2292	0.013
Type of feeding regime	-2.838	-1.419	0.2292	0.000
Adding soil	*4.738*	*2.369*	0.2292**	*0.000*
Interaction of two factors			0.2292	0.000
Type of carbon source × organic loading rate	1.113	0.556	0.2292	0.041
Type of carbon source × type of feeding regime	0.387	0.194	0.2292	0.423
Type of carbon source × adding soil	-3.588	-1.794	0.2292	0.000
*S* = 0.9139	*R*^2^ = 0.9704		*R*^2^_(adj)_ = 0.9445	
**D: 17-alpha-ethynyloestradiol (EE2)**
Constant		81.056	1.228	0.000
Main factors				0.062
Type of carbon source	4.863	2.431	1.228	0.083
Organic loading rate	1.712	0.856	1.228	0.506
Type of feeding regime	-7.513	-3.756	1.228	0.016
Adding soil	1.137	0.569	1.228	0.656
Interaction of two factors				0.110
Type of carbon source × organic loading rate	4.612	2.306	1.228	0.097
Type of carbon source × type of feeding regime	-5.313	-2.656	1.228	0.063
Type of carbon source × adding soil	0.987	0.494	1.228	0.698
*S* = 4.9140	*R*^2^ = 0.7362		*R*^2^_(adj)_ = 0.5054	

The counter intuitive result for resource complexity led us to examine the waste composition in more detail, in particular the energy of the wastes. The complex and the simple wastewater were designed to have the same COD. However, assuming a free energy of 3.75, 3.95, and 4.15 kj/g for monosaccharides (glucose, fructose) for disaccharides (sucrose, lactose,) and starch, respectively, we can see that the free energy of the complex waste provided by the carbohydrates was 1.8 kj/l, while the free energy of the simple waste provided by the carbohydrates was 20% higher, i.e., 2.2 kj/l.

**FIGURE 3:**
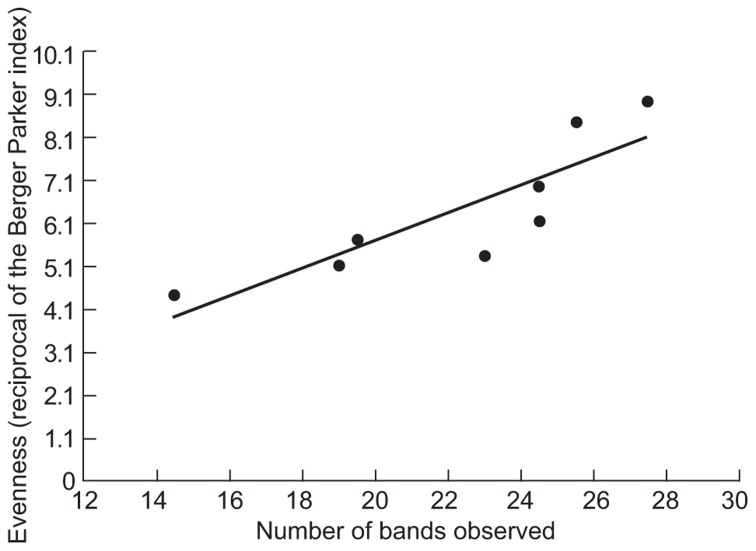
**The relationship between the evenness of the bands on DGGE and the number of the bands**. The Berger–Parker index is the proportional abundance of the most abundant taxon.

The counter intuitive observations of EDC removal in relation to diversity led us to consider the evenness of the DGGE gels. Although DGGE data cannot be considered truly quantitative, we assessed the evenness of the communities in the samples using the Berger–Parker index (the proportional abundance of the most abundant type, based on band intensities). The evenness assessed as the reciprocal of this index (**Figure 3**) was proportional to the number of bands observed (*R*^2^ = 0.74; *p* = 0.006). Having demonstrated that greater observed evenness is associated with greater observed richness we used a simple neutral model to demonstrate how decreasing diversity might increase or decrease the removal of EDCs. Since such compounds were present in concentrations ≤1 μg/l they were not thought to affect the abundance of the taxa responsible for their removal. We can therefore assume that the EDC degraders are present at random at an abundance dictated by their proportional abundance in the metacommunity.

**FIGURE 4:**
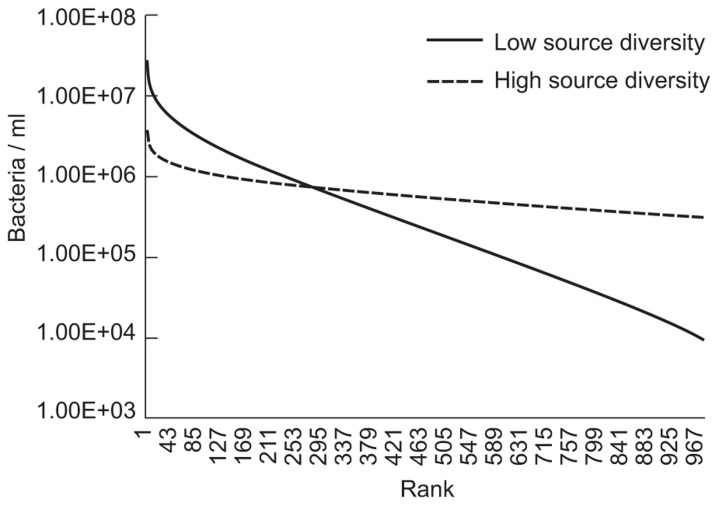
**Results of evenness and diversity using a simple neutral model executed where a source term, or Θ, was set at 176 to illustrate lower diversity and 1760 to illustrate higher diversity**. The immigration parameter (*m*) was set at 3.2 × 10^-9^ and the number of individuals (*N*_*T*_) was set at 1.7 × 10^13^ (equivalent to 17 l with 10^9^ individuals/ml).

The evenness of the microbial community was changed by making arbitrary changes in the size of the source community term. As shown in **Figure 4**, reducing the microbial diversity paradoxically increases the abundance of many taxa that would be considered to be relatively rare.

## DISCUSSION

An appropriately designed statistical trial was unable to unequivocally support simple hypotheses about niche or neutral effects and the relationship between diversity and function. Consequently, the primary conclusion of this paper is that one cannot necessarily use unguided or untested intuition to make inferences about the diversity in microbial communities or how that diversity affects putatively rare functions. However, the failures are potentially more interesting than successes. For failure implies the existence of unsuspected mechanisms and reinforces the importance of experimentation in microbial ecology.

We are confident that the counter-intuitive nature of our findings cannot be attributed to the limitations of DGGE. Fingerprinting methods like DGGE are regarded as inferior to the new generation of sequencing techniques. It is often forgotten that DGGE, like next generation sequencing, uses a large sample size (about 10,000 individuals). Studies with next generation sequencing have suggested this sample size is enough to make valid comparisons between datasets. The weakness of DGGE is that it can only detect those taxa that occur above an abundance threshold of about 1% of the community under consideration (Muyzer et al., 1993). The implicit assumption in this study is that a change in diversity is associated with a change in the number of taxa above this threshold. The association between evenness and the number of taxa detected supports this assumption. Moreover, some large-scale patterns first detected with fingerprinting methodologies (Fierer and Jackson, 2006) have been found to be substantially unaltered when re-examined with larger datasets (Lauber et al., 2009).

Thus it is gratifying, but not interesting, that creating temporal variation (using a SBR) and increasing the number of individuals (by increasing the organic load) both appeared to increase diversity. Both results seem intuitively reasonable from a niche and neutral perspective, respectively. That both perspectives appear valid strengthens our assertion, supported by recent modeling work, that niche effects will overlay neutral ones (Ofiteru et al., 2010). However, it is this relatively bland interpretation that emphasizes the challenge and importance of attempting to quantify the relative importance of these mechanisms, which we are unable to do with data in this format.

The failure of immigration to increase diversity is not, on reflection, surprising. The organisms in the soil were at or below the detection limit for DGGE and so will not have been directly detectable unless they increased in abundance in the system. Ecologists recognize that it is difficult to “invade” mature communities and have formulated the monopolization hypothesis to account for this phenomenon (De Meester et al., 2002). There is evidence that immigration occurs at low rates in microbial communities subject to high rates of dispersal (Curtis et al., 2006; Baptista et al., in preparation). This phenomenon may well underlie the poor reputation of bio-augmentation in many quarters. The slight drop in the diversity associated with the addition of soil is harder to interpret from a neutral perspective. There are possible niche-based explanations. For example, the decreased diversity in the glucose treatment could be a result of competition from fewer, but more competitive species from the soil. Alternatively, it might be attributable the small quantity of sodium cholate used to suspend the soil.

However, the failure of a feed with a more complex chemical composition to support greater diversity is problematic. We do not believe this is an artifact, as we have also recently failed to increase diversity by increasing resource complexity in a bioelectrochemical cell (Heidrich, submitted). We are unable to conceive of a simple niche-based explanation though we admit that multiple resource can have complex effects (Lendenmann and Egli, 1998). A NCM requires that we invoke either a more diverse source community, higher rate of migration or a larger local community. We have no reason to invoke a higher immigration rate in glucose fed reactors. However, the biomass was slightly and significantly (Pholchan et al., 2010) higher with glucose (which has a slightly higher free energy) than with complex wastes, even though the COD of the feeds were the same. This suggests that the yields are higher for organisms growing on glucose than on complex wastes. The larger amount of biomass could lead to a higher diversity. However, we have previously postulated that groups of organisms with higher yields will also have higher evolution rates and thus higher source diversities (Curtis et al., 2008). To separate these two possibilities it would be necessary to fit a neutral model to our findings. This is not possible with the data we have generated.

To interpret the ostensibly contradictory and counterintuitive findings with respect to diversity and EDC removal, we must recall that the functions rendered by a microbial community will depend on the abundance of the organisms performing that function and the environment encountered. It is clear from the simple community assembly model that a drop in evenness in a microbial community, which would be associated with a drop in observed richness, could also lead to an increase in the abundance of certain taxa. This would explain why EDC removal increased when diversity decreased in reactors fed with complex wastes or dosed with a soil suspension. The corollary that increases in diversity lead to a reduction in EDC removal was (i) perceptible at higher organic loading rates where the removal of E3 was lower (with no effect being seen with E1, E2, or EE2) but (ii) absent in higher diversity SBRs. However, SBRs are effectively plug flow reactors and thus far more hydraulically efficient than CSTRs. Consequently, the former (SBRs) may achieve higher effluent quality than the latter (CSTRs), even with an inferior removal rate.

We conclude that it is not possible to make simple blanket statements about the relationship between putative rare functions and diversity. This supports earlier insightful suggestion that ecologists should not always assume that increased biodiversity always means improved function (Jiang et al., 2008) and warn against extending findings on biomass and diversity (Tilman et al., 1997) to other functions.

If our interpretation is correct, this relationship may well depend on the proportional abundance of the organisms with that function in the metacommunity, the size of the local community and the immigration rates that connect the former and the latter. It will thus be predictable using neutral theory, but not intuitively obvious and, of course, subject to other environmental constraints. The application of this approach would require that we eschew the nebulous term rare and define the proportional abundance, and possibly activity, of the taxa responsible for the removal of a given micropollutant. This is a study of a series of pilot plants seeded from just one “mother” plant and we cannot be sure how generalizable our findings are. However, neutral theory (if applicable) would allow us to determine “a priori” how generalizable our findings by relating the probability of a function occurring to the proportional abundance of the organisms with that function in the metacommunity.

The niche and neutral perspectives are not mutually exclusive. One might also explain the patterns in these relatively rare functions by invoking the appearance and disappearance of niches for the EDC degrading organisms. However, we have no evidence to support this perspective which, if true, could mean the presence and absence of functions is situation bound.

In summary we suggest that, in this system, an increase in evenness equates with a decrease in abundance of some abundant taxa and an increase in abundance in some rare taxa. This is why a change in evenness can lead to a decrease in the removal of some EDC and an increase in others. Unless and until we know the proportional abundance of the EDC removing taxa we will not be able to determine the effect of diversity on EDC removal. Unless we know the mechanism governing the diversity (niche or neutral or both) we will not be able to predict EDC removal.

## Conflict of Interest Statement

The authors declare that the research was conducted in the absence of any commercial or financial relationships that could be construed as a potential conflict of interest.
